# Maresin Biosynthesis and Identification of Maresin 2, a New Anti-Inflammatory and Pro-Resolving Mediator from Human Macrophages

**DOI:** 10.1371/journal.pone.0102362

**Published:** 2014-07-18

**Authors:** Bin Deng, Chin-Wei Wang, Hildur H. Arnardottir, Yongsheng Li, Chien-Yee Cindy Cheng, Jesmond Dalli, Charles N. Serhan

**Affiliations:** Center for Experimental Therapeutics and Reperfusion Injury, Department of Anesthesiology, Perioperative and Pain Medicine, Brigham and Women's Hospital, Harvard Medical School, Boston, Massachusetts, United States of America; McMaster University, Canada

## Abstract

Maresins are a new family of anti-inflammatory and pro-resolving lipid mediators biosynthesized from docosahexaenoic acid (DHA) by macrophages. Here we identified a novel pro-resolving product, 13R,14S-dihydroxy-docosahexaenoic acid (13R,14S-diHDHA), produced by human macrophages. PCR mapping of 12-lipoxygenase (12-LOX) mRNA sequence in human macrophages and platelet showed that they are identical. This human 12-LOX mRNA and enzyme are expressed in monocyte-derived cell lineage, and enzyme expression levels increase with maturation to macrophages or dendritic cells. Recombinant human 12-LOX gave essentially equivalent catalytic efficiency (k_cat_/K_M_) with arachidonic acid (AA) and DHA as substrates. Lipid mediator metabololipidomics demonstrated that human macrophages produce a novel bioactive product 13,14-dihydroxy-docosahexaenoic acid in addition to maresin-1, 7R,14S-dihydroxy-4Z,8E,10E,12Z,16Z,19Z-docosahexaenoic acid (MaR1). Co-incubations with human recombinant 12-LOX and soluble epoxide hydrolase (sEH) demonstrated that biosynthesis of 13,14-dihydroxy-docosahexaenoic acid (13,14-diHDHA) involves the 13S,14S-epoxy-maresin intermediate produced from DHA by 12-LOX, followed by conversion via soluble epoxide hydrolase (sEH). This new 13,14-diHDHA displayed potent anti-inflammatory and pro-resolving actions, and at 1 ng reduced neutrophil infiltration in mouse peritonitis by ∼40% and at 10 pM enhanced human macrophage phagocytosis of zymosan by ∼90%. However, MaR1 proved more potent than the 13R,14S-diHDHA at enhancing efferocytosis with human macrophages. Taken together, the present findings demonstrate that macrophages produced a novel bioactive product identified in the maresin metabolome as 13R,14S-dihydroxy-docosahexaenoic acid, from DHA via conversion by human 12-LOX followed by sEH. Given its potent bioactions, we coined 13R,14S-diHDHA maresin 2 (MaR2).

## Introduction

The acute inflammatory response is the host protection against harmful stimuli, including pathogen infections, irritants and cell damage [1]. New families of lipid mediators, derived from n-3 polyunsaturated fatty acid (PUFA), that are biosynthesized during the resolution phase of acute inflammation and coined resolvins and protectins, were uncovered that potently stop inflammation promoting catabasis and return to homeostasis [2]. Macrophages play a central role in resolution, wound healing and tissue repair [3]. Maresins, macrophage mediators in resolving inflammation, are a family of lipid mediators biosynthesized by macrophages from DHA via human 12-lipoxygenase (12-LOX) [4]. Maresin 1 (7R,14S-dihydroxy-docosa-4Z,8E,10E,12Z,16Z,19Z-hexaenoic acid or MaR1), the first member of this family identified, displays potent anti-inflammatory and pro-resolving actions inhibiting neutrophil infiltration *in vivo*, and stereoselectively stimulating macrophage phagocytosis and efferocytosis [4], [5]. The complete stereochemistry of MaR1 has been established, and MaR1 also displays potent tissue regenerative as well as anti-nociceptive actions [5].

Biosynthesis of maresins in macrophages involves an initial oxygenation of DHA with molecular oxygen [4], followed by epoxidation of the 14 hydroperoxy-intermediate that is subsequently converted to 13S,14S-epoxy-maresin [6]. Evidence for 13S,14S-epoxy-maresin was recently obtained from both human macrophages [4] and recombinant human 12-LOX incubation [6] using alcohol trapping. The complete stereochemistry of this epoxide intermediate was determined, 13S,14S-epoxy-docosa-4Z,7Z,9E,11E,16Z,19Z-hexaenoic acid, by total organic synthesis as well as its biological actions were uncovered [6].

Given the potent pro-resolving actions of MaR1 [4], [5], it remained of interest to characterize the role of human 12-LOX in macrophages in maresin biosynthesis, and whether 13S, 14S-epoxy-maresin intermediate is converted to additional bioactive products by human macrophages. Here, we characterized the human macrophage 12-LOX and its role in maresin biosynthesis, and identified a new bioactive macrophage product (13R,14S-dihydroxy-4Z,7Z,9E,11E,16Z,19Z-hexaenoic acid) coined MaR2 that displays anti-inflammatory and pro-resolving actions.

## Materials and Methods

### Materials

RPMI 1640 and DPBS (with or without calcium and magnesium) were purchased from Lonza (Hopkinton, MA, USA). Ficoll-Histopaque 1077-1, lipopolysaccharide (LPS) and zymosan A were obtained from Sigma-Aldrich (St. Louis, MO, USA). Human recombinant granulocyte-monocyte colony stimulating factor (GM-CSF), macrophage colony simulating factor, interferon γ (IFN-γ) and interleukin-4 were purchased from R&D System (Minneapolis, MN, USA). Fetal calf sera, ampicillin and bac-to-bac baculovirus expression system were from Invitrogen (Grand Island, NY, USA). High capacity cDNA reverse transcription kit was from Applied Biosystems (Grand Island, NY, USA). Phusion PCR kit and cloning enzymes were from New England BioLabs (Ipswich, MA, USA). All primers were synthesized by Integrated DNA Technologies (Coralville, IA, USA). Bio-scale Mini Macro-prep High Q columns were from BioRad (Hercules, CA, USA). Sephacryl S-100 HR resin was from GE Life Sciences (Pittsburgh, PA, USA). FACS analysis antibodies against the surface marks were from BioLegend (San Diego, CA, USA). FITC rat anti-mouse Ly6G (clone IAS), purified rat anti-mouse CD16/32 (mouse BD Fc block) were purchased from BD Bioscience (San Jose, CA), and PE rat anti-mouse F4/80 (clone BM8) and PerCPCy5.5 rat anti-mouse CD11b (clone Mac-1) were purchased from eBioscience (San Diego, CA). Human 12-lipoxygenase monoclonal antibody was from Novus Biologicals (Littleton, CO, USA). Human soluble epoxide hydrolase polyclonal antibody, soluble epoxide hydrolase, AA, DHA, d_8_-5S-HETE and d_4_-LTB_4_ purchased were from Cayman Chemicals (Ann Arbor, MI, USA). Human soluble epoxide hydrolase shRNA constructs were from OriGene (Rockville, MD, USA). C18 SPE columns were from Waters (Milford, MA, USA). Eclipse Plus C18 column was from Agilent (Santa Clara, CA, USA). All liquid chromatography solvents were from Fisher Scientific (Pittsburgh, PA, USA).

### Human macrophage and dendritic cell preparation

Human peripheral blood mononuclear cells (PBMC) from whole blood (purchased from Children's Hospital Blood Bank, Boston, MA, de-identified from healthy donors) were isolated by density gradient centrifugation using Histopaque-1077. Monocytes (MC) were obtained by adhesion purification, and were cultured in RPMI with 10 ng/mL human recombinant GM-CSF at 37°C for 7 days to obtain macrophages (M0). M1 macrophages were obtained by stimulating M0 macrophages with 20 ng/ml IFN-γ and 1 ng/ml LPS for 24 h. M2 macrophages were obtained by incubating monocytes with 20 ng/ml macrophage colony simulating factor for 6 days and stimulating with 20 ng/ml IL-4 for 48 h [7]. Immature dendritic cells (iDC) were obtained by incubating isolated MCs with 50 ng/ml GM-CSF and 34 ng/ml IL-4 for 7days, and mature dendritic cells (mDC) were stimulated by 100 ng/ml LPS for 24 h [8]. Phenotypic lineage of monocyte-derived cells was confirmed by flow cytometry staining with fluorescent-conjugated antibodies, including CD163, CD54 and CD80 [7].

### Quantitative real-time PCR (qPCR)

Total RNA was isolated from cultured cells using High Pure miRNA Isolation Kit (Roche), and cDNA was synthesized with High Capacity Reverse Transcription Kit (Applied Biosystems) according to the manufacturer's protocol. Real-time PCR was performed using the SYBR-green [9]. GAPDH was employed as internal control. Expression was measured on an ABI Prism cycler (Applied Biosystems, Foster City, CA) and data were analyzed using the ΔΔCt method. The forward and reverse primers used were as follows: 12-LOX (5′-GATGATCTACCTCCAAATATG-3′ and 5′-CTGGCCCCAGAAGATCTGATC-3′) and GAPDH (5′-AGCCACATCGCTCAGACAC-3′ and 5′-GCCCAATACGACCAAATCC-3′).

### Cloning of human macrophage 12-lipoxygenase

12-LOX cDNA was synthesized with high capacity cDNA reverse transcription kit (Applied Biosystems) and a 12-LOX specific RT primer from M0 macrophage total mRNA. The primer sequence is 5′-AGA AAG TTT ACT GCT CCC CTG G-3′. Two pairs of primers were designed to amplify the upstream and downstream regions. The two regions cover the whole coding area of 12-LOX cDNA and had ∼200 bp overlap. The upstream primer sequences were: 5′-CTC CCC TCG CCT AAG CTG CTG-3′; 5′-CTT GAA GAT GGG GTG CAG TCC-3′. The downstream primer sequences are: 5′-ATT CAG CCT CCC AAC CCC AGC TCT-3′; 5′-GGT TTA ACT GGG GGA GGA AAT AGA GCC T-3′. PCRs were performed with Phusion PCR kit (New England BioLabs) following manufacturer's instruction to amplify the upstream and downstream regions. Human 12-LOX full-length cDNA coding 663 amino acid residues was obtained by using the obtained upstream-downstream PCR products as template, and performing a second round of PCR (SOEing PCR). NdeI and BamHI restriction sites were included in front of start codon and after stop codon, respectively. 12-LOX full-length cDNA was inserted into pET20b vector and sequenced.

### Expression of human macrophage 12-LOX protein in sf9 cells

12-LOX protein was obtained with Bac-to-Bac Baculovirus Expression System (Invitrogen) and the manufacturer's instructions. In brief, 12-LOX cDNA was sub-cloned into pFastBac vector. pFastBac-12-LOX plasmid was transformed into DH10Bac *E. coli*, and was recombined with bacmid. The fragment with 12-LOX was donated into bacmid. The bacmid harboring 12-LOX was isolated and transfected into insect sf9 cells. After 5 days, the first generation virus (P1) was harvested from the media. 0.5 to 1 ml of P1 was used to infect 10 ml of sf9 cells to obtain the second generation of virus (P2). 125 ml of sf9 cells with density of 2×10^6^ cells/ml in suspension culture were infected with 10 ml P2 at 27°C for 72 h. Cells were stored at −80°C after centrifugation at 2000 g for 10 min.

### Protein purification

Sf9 cells were suspended in 50 mM Tris-HCl (pH 8) buffer supplemented with protease inhibitor cocktail (sigma), and disrupted by sonication (Branson, 20 s pulse for 5 times). Soluble fraction was isolated by centrifugation at 15000 g for 30 min for column chromatography. 12-LOX protein was purified to homogeneity by ion exchange and size exclusion column chromatography consecutively using Bio-scale Mini Macro-prep High Q (5 ml) and Sephacryl S-100 HR (80 ml) columns at a flow rate of 1.0 and 0.35 ml/min, respectively. For Q column, a linear gradient between 0 and 1 M NaCl in 50 mM Tris-HCl (pH 8) was used to elute all proteins in 20 column volumes. The fractions with 12-LOX activity (see next section) were collected, pooled, concentrated and loaded onto Sephacryl S-100 HR column with 50 mM Tris-HCl (pH 8). All procedures were conducted on ice or at 4°C. Protein purity was determined to be about 90% by SDS-PAGE. Small aliquots of concentrated human recombinant 12-LOX were flash frozen in liquid nitrogen and stored at −80°C until use.

### Kinetic assays

Assays were performed using a Cary 60 UV-Vis spectrophotometer. Either AA or DHA from 1 µM to 50 µM was used as substrate [S], and 12-LOX concentration [E] was fixed at 100 nM. Assays were conducted either in calcium-depleted buffer (50 mM Tris, pH 8, 1 mM EGTA, 2 mM MgCl_2_, 0.03% Tween 20) or in calcium-supplemented buffer (50 mM Tris, pH 8.0, 1 mM EGTA, 2 mM MgCl_2_, 3 mM CaCl_2_, 0.03% Tween 20). Conversion of AA to HpETEs or DHA to HpDHA was monitored by UV absorbance at 235–236 nm. At each given substrate concentration, initial rate (V_0_) was obtained using extinction coefficient ε_236_≈23,000 M^−1^cm^−1^. V_0_ and substrate concentration [S] were fit into Michaelis-Menten equation: V_0_ = [E]×k_cat_×[S]/(K_M_+[S]) to determine K_M_ and k_cat_.

### Mouse peritonitis

All animal procedures were approved by the Standing Committee on Animals of Harvard Medical School (Protocol 02570) and performed in accordance with institutional guidelines. Male FVB mice (6–8 weeks old, weighing 22–25 g) were purchased from Charles River Laboratories. Mice were housed 4 mice per cage in specific pathogen-free facilities in a humidity (45–55%) and temperature (23–25°C) controlled environment with a 12 h light-dark cycle. Mouse peritonitis was carried out as in [10] and efforts were made to minimize suffering (e.g. if an increase in pain or stress behavior was noted, the mice were euthanized according to Protocol #02570). Briefly, mice were anesthetized by inhaled isofluorane according to Protocol 02570 and randomly assigned to be administered intravenously with 1 ng 13R,14S-diHDHA, MaR1, or vehicle (saline) immediately prior to the administration of 0.1 mg zymosan [11]. At 4 h, mice were euthanized with overdose of isoflurane followed by cervical dislocation and peritoneal lavages collected. PMN numbers were assessed by light microscopy and flow cytometry. PMNs were identified as CD11b^+^ and Ly6G^+^ cells.

### Human macrophage phagocytosis

Human M0 macrophages were plated onto 96-well plates (5×10^4^ cells/well) and phagocytosis was carried out after 24 h. Cells were incubated with either vehicle (PBS containing 0.1% ethanol), MaR1 as in [4], [5] or 13R,14S-diHDHA at indicated concentrations (15 min, 37°C). All incubations were conducted in PBS containing calcium and magnesium (PBS^+/+^). Subsequently either FITC-labeled zymosan (5×10^5^ particles/well) or fluorescent labeled apoptotic PMN (1.5×10^5^ cells/well) were added to each well and cells incubated for a further 60 min at 37°C, pH 7.45. The cells were washed 3 times with PBS^+/+^ and extracellular fluorescence was quenched using Trypan Blue (1∶15 diluted). Phagocytosis was assessed using a SpectraMax M3 plate reader (Molecular Devices).

### Human recombinant enzyme incubations

To identify the chirality of 12-LOX product(s), DHA (5 µM) was mixed with the isolated human macrophage 12-LOX (0.2 µM) in 100 µL 20 mM Tris (pH 8), 100 mM KCl at 37°C for 10 min. In select incubations, DHA (10 µM) was either incubated with human 12-LOX or with 12-LOX plus sEH (0.28 µM) in the same Tris buffer as above at 37°C, pH 8.0 for 10 min. All incubations were stopped by 2 volumes of methanol, and 500 pg d_8_-5S-HETE and d_4_-LTB_4_ were added into each sample before extraction as internal standards [12].

### Sample extractions and LC-MS/MS

All samples from recombinant enzyme incubations were subjected to liquid-liquid extractions with 6 volumes of diethyl ether. The samples from cell incubations were extracted with SPE columns [12]. LC-MS/MS analyses were conducted as in [6]. In the chiral chromatography separation, Chiralpak AD-RH column was used with methanol/water/acetic acid of 95∶5∶0.01 (v/v/v) [13]. The transition ion pairs were: 14-hydroxy-4Z,7Z,10Z,12E,16Z,19Z-docosahexaenoic acid (14-HDHA) (343>205) and 13,14-diHDHA (359>221). 500 pg d_8_-5S-HETE and d_4_-LTB_4_ were used as internal standards to determine extraction recoveries.

### Western blotting

The procedure was modified from [14]. In brief, cell lysis was mixed with equal volume of tricine sampling buffer (BioRad), and mixtures were immediately boiled at 95°C for 4 min. The samples were loaded to 10% SDS-PAGE, and was transferred to nitrocellulose membranes after gel separation. Membranes were probed by primary and secondary antibodies consecutively. Primary antibodies were diluted as instructions: 1∶500 for anti-human 12-LOX (Novus Biologicals); 1∶200 for anti-human sEH (Cayman); 1∶200 for anti β-actin (Santa Cruz). Blots were developed by enhanced chemiluminescence (Pierce).

### Statistics

All the results were expressed as means ± standard error. One-way ANOVA was carried out to determine difference between groups. *P*<0.05 was considered significant.

## Results

### Human monocyte-derived lineage express 12-LOX

**Figure 1:**
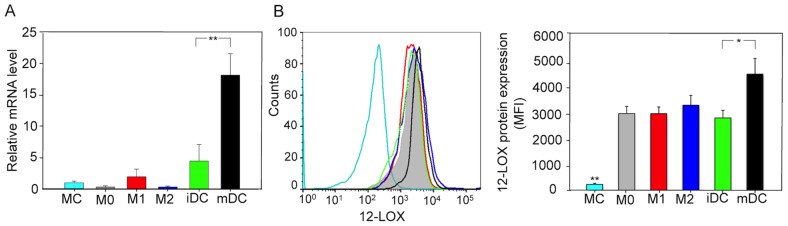
Human macrophages and dendritic cells express human 12-LOX. (A) Expression levels of 12-LOX mRNA in human monocytes (MC), M0, M1, M2 macrophages, immature dendritic cells (iDC) and mature dendritic cells (mDC) were assessed by quantitative PCR. (B) 12-LOX protein expression levels were assessed by flow cytometry. In the left panel, a representative histogram shows 12-LOX present in each cell type. In the right panel, results are mean fluorescent intensity (MFI) of 12-LOX protein levels for each cell type, expressed as mean±SEM of 4 separate cell preparations (*P<0.05, **P<0.01).

We first examined 12-LOX expression levels in human monocytes (MC), macrophages (M0, M1 and M2) and dendritic cells (DC). The cells were prepared in accordance with published methods [7], [8]. Quantitative PCR results using the human platelet 12-LOX sequences as primers (see methods) gave rather low 12-LOX mRNA levels in MC, M0, M1 and M2 cells as well as iDCs (immature dendritic cells) (**Fig. 1a**). In contrast, the 12-LOX mRNA level in mDC (mature dendritic cells) was significantly higher, or about 3.5 fold compared to iDC. Also, flow-cytometry assessment using fluorescence-labeled antibody against human 12-LOX demonstrated about 10 fold increase in protein expressions in M0, M1 M2 and iDC, and 19 fold increase in mDC, compared to MC (**Fig. 1b**), indicating that expression of human 12-LOX protein is enhanced with differentiation of MC to macrophages and dendritic cells. Cell lineage was determined by flow cytometry using distinct phenotypic markers (CD80 for M1, CD163 for M2, CD54 for DC, **Fig. S1**) [7].

We amplified 24-1214 and 1005–2167 bp fragments of 12-LOX from human macrophages (M0) cDNA (**Fig. S2**). The two fragments covered the entire coding area, and had about 200 bp overlapping. We numbered nucleotides in the present report on the basis of the platelet cDNA sequence [15]. We obtained the full length of human macrophage 12-LOX cDNA by SOEing PCR with the 24–1024 and 1005–2167 fragments as template, and the sequencing results showed that M0 12-LOX cDNA sequence was in accordance with that reported earlier for human platelet 12-LOX [15] (see **Fig. S2**). Also, western blotting for 12-LOX revealed 75 kD size bands in macrophages and dendritic cell, which is consistent with the size of human platelet 12-LOX (**Fig. S2**).

### Human macrophage recombinant 12-LOX converts DHA and AA with essentially equivalent catalytic efficiency

**Figure 2:**
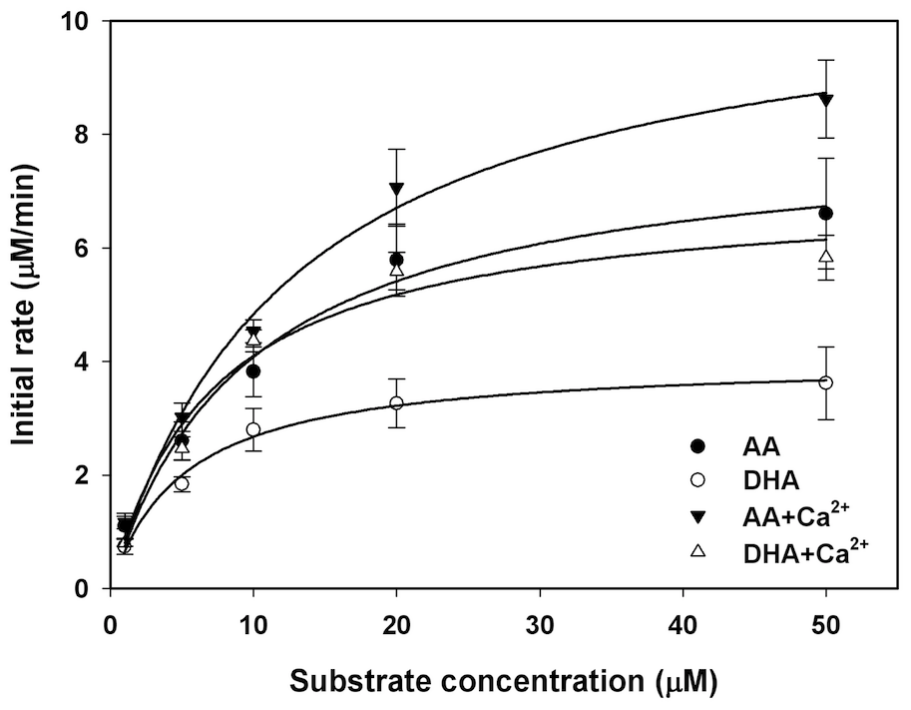
Human 12-LOX converts AA and DHA with essentially equivalent efficiency. Increasing concentrations of AA or DHA (1 to 50 µM) were mixed with 12-LOX (0.1 µM, pH 8.0, R.T.) in the presence or absence of CaCl_2_ (2 mM). Initial rates were monitored and plotted versus substrate at indicated concentrations. Each point represents mean±SEM from n = 3 separate experiments.

We next examined and compared the catalytic efficiencies of AA and DHA with human macrophage recombinant 12-LOX. AA or DHA were incubated with 0.1 µM human recombinant macrophage 12-LOX in the presence or absence of CaCl_2_. The initial rate at each substrate concentration was plotted and fit to Michaelis-Menten equation using non-linear regression to determine k_cat_ (maximal turnover rate per enzyme) and K_M_ (concentration of substrate with initial rate reaching half of maximal rate, **Fig. 2** **,** **Table 1**). The calculated k_cat_ for DHA was 2 fold lower than AA; however, the calculated K_M_ for DHA was about 2 fold lower than that of AA, suggesting that the maximal turnover of DHA by 12-LOX is slower, while affinity of DHA to 12-LOX is higher when compared to AA. The resulting catalytic efficiencies, indicated by k_cat_/K_M_, were essentially equivalent between DHA and AA. Also, addition of CaCl_2_ appeared to increase both k_cat_ and K_M_, yet lead to no significant increase in catalytic efficiency (k_cat_/K_M_) (**Table 1**).

**Table 1:** Parameters of Michaelis-Menten kinetics.*

	K_M_ (µM)	k_cat_ (min^−1^)	k_cat_/K_M_ (min^−1^ µM^−1^)
AA	9.7±2.1	80.3±6.0	8.3
DHA	5.1±0.7	40.4±1.6	7.9
AA+Ca^2+^	12.6±2.2	109.4±7.1	8.7
DHA+Ca^2+^	7.1±2.0	70.2±6.1	9.9

*Initial rate at each substrate concentration was fitted into Michaelis-Menten equation to determine K_M_ and k_cat_. Results are mean ± SEM.

### Stereo and positional selective production of 14S-HpDHA by human macrophage 12-LOX

**Figure 3:**
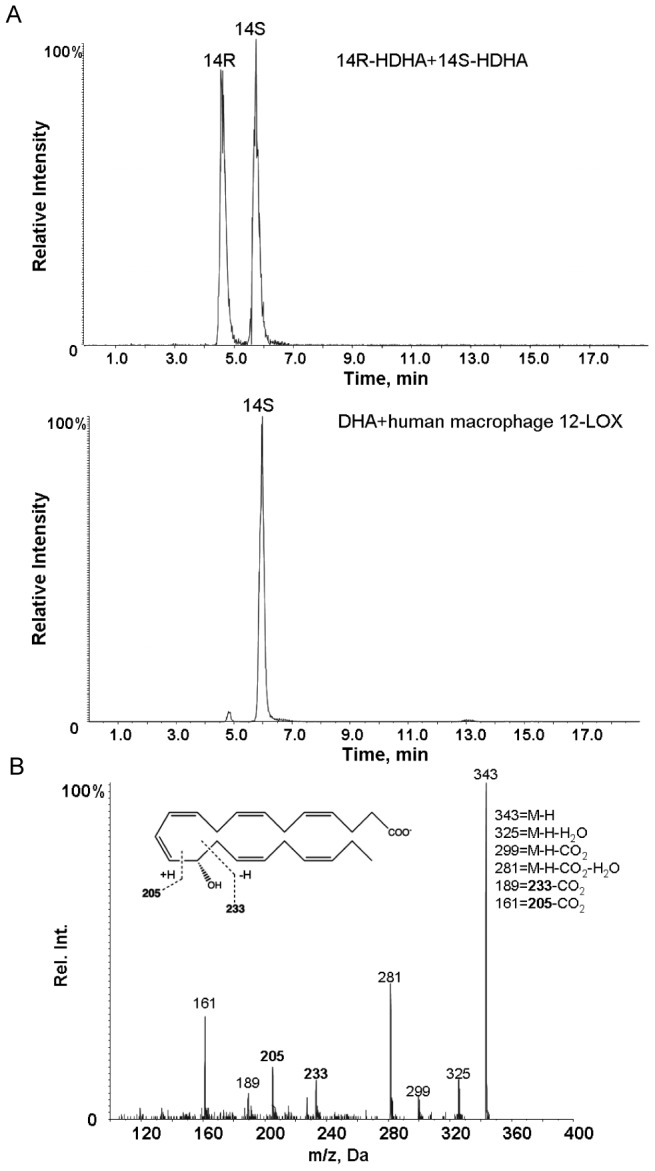
Human macrophage 12-LOX produces 14S-HpDHA. (A) Chiral chromatography of synthetic 14R-HDHA and 14S-HDHA (upper panel). DHA (5 µM) was incubated with human macrophage 12-LOX (0.2 µM, 10 min, 37°C, pH 8). Products were extracted (see methods for details) and subject to chiral high performance liquid chromatography-tandem mass spectrometry (m/z, 343>205) (lower panel). (B) MS-MS spectrum of 14S-HDHA from human macrophage 12-LOX incubations (lower panel in A).

Recombinant human macrophage 12-LOX was expressed with baculovirus-insect system, and was purified to apparent homogeneity as described in methods. DHA was incubated with human macrophage 12-LOX. After reduction of reaction mixture using NaBH_4_, the hydroxyl-containing products were separated using chiral high performance liquid chromatography and identified using lipid mediator metabololipidomics. We found that in these incubations the peak at retention time of ∼6 min accounted for ∼98% peak area and matched the retention time of 14S-HDHA (**Fig. 3a**). The MS-MS spectrum for the product beneath this peak matched that of 14-HDHA (**Fig. 3b**). These results demonstrate that human macrophage 12-LOX inserted oxygen at the C-14 position of DHA predominantly in the S conformation.

### Identification of endogenous 13,14S-diHDHA from human macrophages

**Figure 4:**
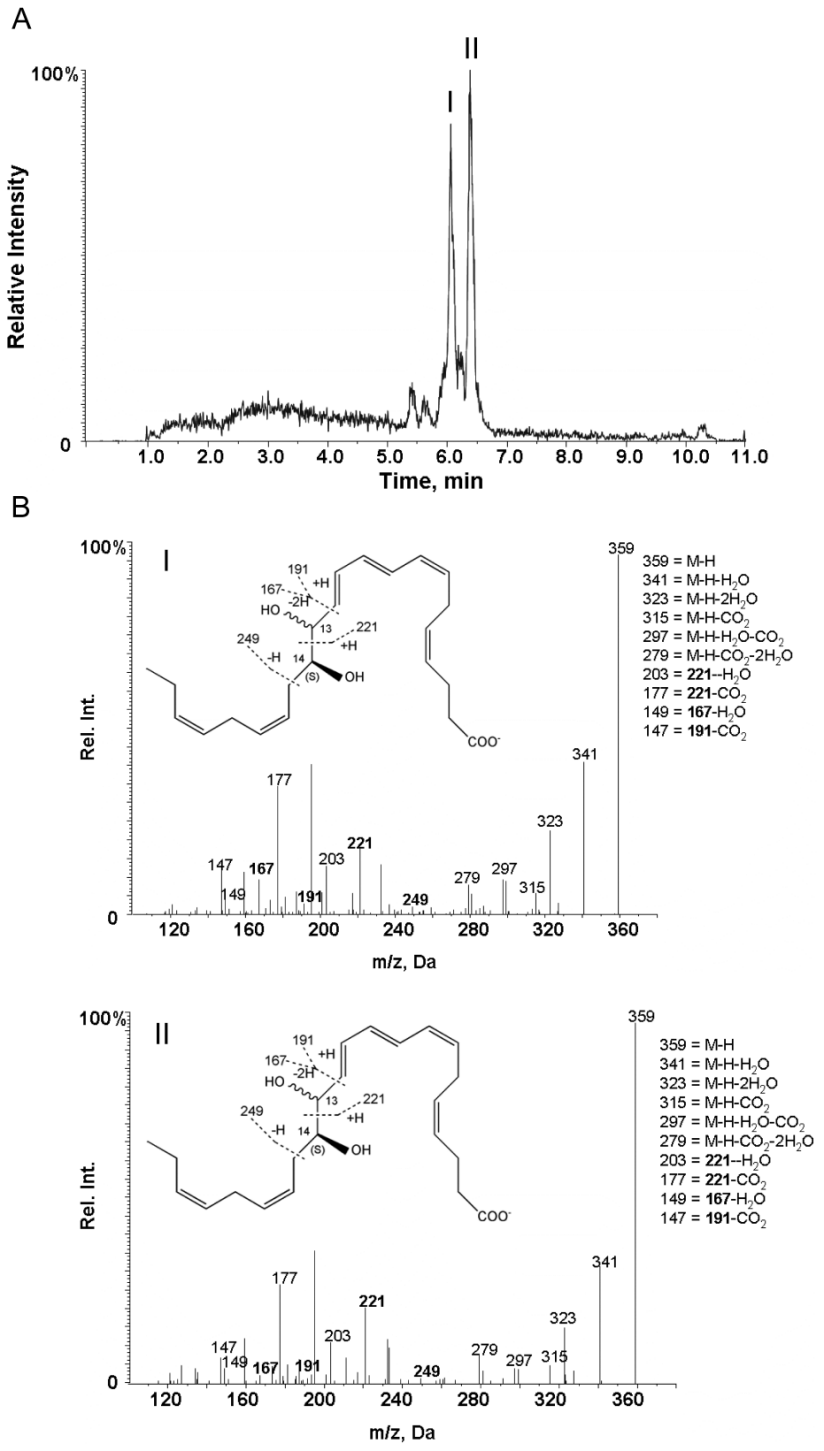
Human macrophage endogenous production of 13R,14S-diHDHA. (A) MRM chromatography for ion pair 359>221 (m/z). Human macrophages were incubated with opsonized zymosan (DPBS+/+, pH 7.45, 15 min, 37°C). Incubations were stopped with 2 volumes of ice-cold methanol, and products were assessed by lipid mediator metabololipidomics. (B) MS-MS spectra. *Inset*, diagnostic ions used for identification and corresponding possessed structure fragments. Results are representative of n = 3.

We next investigated whether the 13S,14S-epoxy-maresin, a biosynthetic product of human macrophage 12-LOX [6], was converted by human macrophage to novel products. Using lipid mediator metabololipidomics, we identified 2 endogenous products from macrophages with the fragmentation spectrum for the product under peak I matching 13,14S-diHDHA with the following ions assigned: *m/* *z* 359 = M-H, *m/* *z* 341 = M-H-H_2_O, *m/* *z* 323 = M-H-2H_2_O, *m/* *z* 315 = M-H-CO_2_, *m/* *z* 297 = M-H-H_2_O-CO_2_, *m/* *z* 279 = M-H-2H_2_O-CO_2_, *m/* *z* 203 = 221-H_2_O, *m/* *z* 177 = 221-CO_2_, *m/* *z* 149 = 167-H_2_O, and *m/* *z* 147 = 191-CO_2_ (**Fig. 4**). Peak II with retention time of 6.5 min displays the essentially identical fragmentation to peak I, and thus suggests that the product beneath peak II was a stereoisomer of peak I.

### Cell-free biosynthesis of 13,14S-HDHA

**Figure 5:**
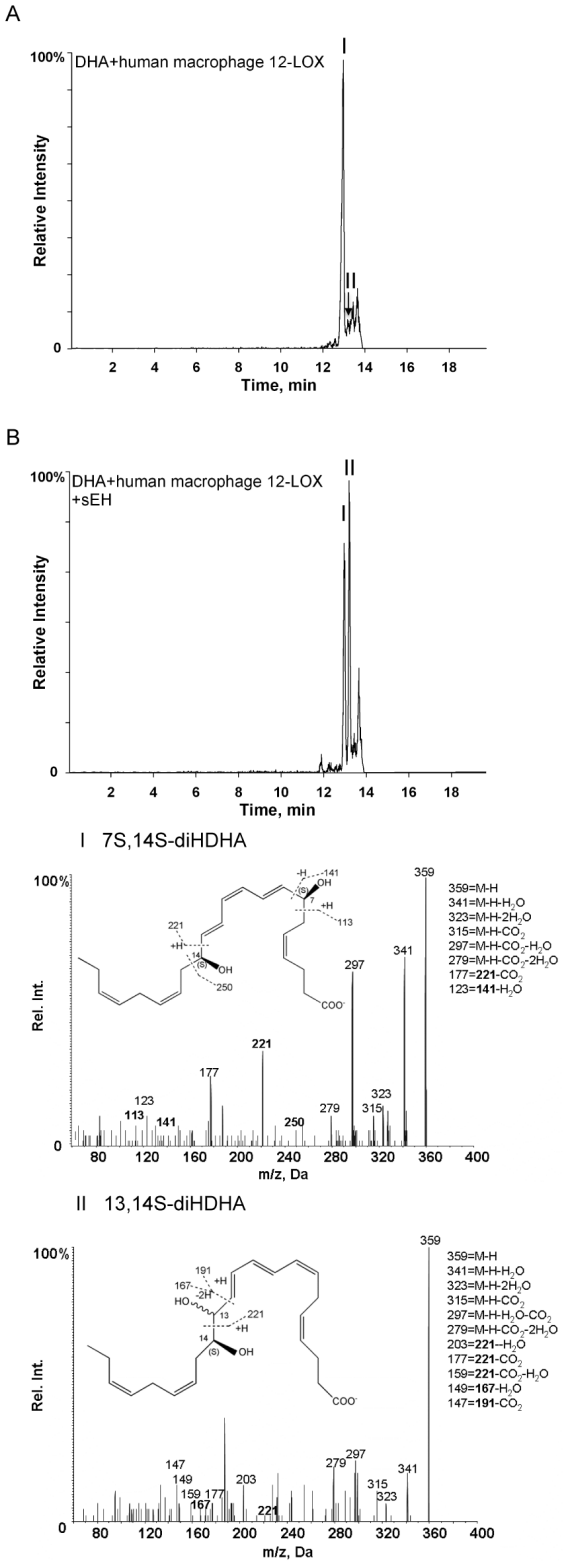
Biosynthesis of 13R,14S-diHDHA in coincubations with human recombinant 12-LOX and sEH. (A) MRM chromatography for ion pair 359>221. DHA (10 µM) was incubated with 12-LOX (0.2 µM) in the absence or presence of 2 U sEH (20 mM Tris, pH 8.0, 100 mM KCl, 37°C, 10 min). (B) MS/MS spectra employed in the identification of 13,14S-diHDHA (peak II) and 7S,14S-dihydroxy-4Z,8E,10Z,12E,16Z,19Z-docosahexaenoic acid (7S,14S-diHDHA) (peak I). Results are representative of n = 3.

sEH is expressed in cells of the monocyte lineage [16], [17]. sEH converts leukotriene A_4_ to 5S,6R-diHETE [18] and epoxyeicosatrienoic acids to vicinal diols [19]. Therefore, we next investigated whether this enzyme was involved in the biosynthesis of 13,14S-diHDHA. Co-incubation of both human macrophage 12-LOX and sEH with DHA gave 13,14S-diHDHA (peak II, ∼0.1% conversion) in comparison to scant formation of this diol product (∼0.005% conversion) when only DHA and 12-LOX were present in the incubations (**Fig. 5a**). The MS-MS fragmentation of 13,14S-diHDHA (peak II, **Fig. 5b**) using this recombinant enzyme system matched those obtained from macrophages (**Fig. 4**). In the incubations with 12-LOX and DHA, we also identified a double oxygenation product (7S, 14S-diHDHA; peak I, **Fig. 5b**), whereas, MaR1 was not identified in these coincubations. These results suggest that 13,14-epoxy-maresin [6] produced from DHA via 12-LOX is substrate of sEH producing 13,14S-diHDHA.

### 13,14S-diHDHA displays anti-inflammatory and pro-resolving bioactions

**Figure 6:**
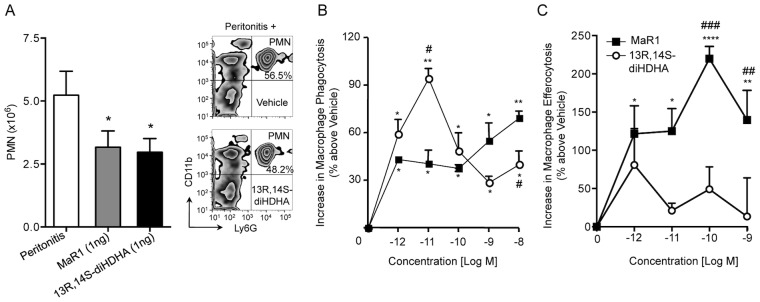
MaR1 and MaR2 (13R,14S-diHDHA) display potent anti-inflammatory and proresolving actions: direct comparison. (A) Mouse peritonitis: exudate PMN numbers *in vivo*. Male mice (6–8 weeks) were administered *i.v*. MaR1, 13R,14S-diHDHA (1 ng/mouse each) or vehicle prior to *i.p*. administration of zymosan (0.1 mg/mouse). Peritoneal exudates were collected, and PMNs enumerated using both light microscopy and flow cytometry. Results are mean ± SEM. n = 3 mice per treatment from three separate experiments (*P<0.05 *vs*. vehicle). Enhanced phagocytosis of (B) opsonized zymosan or (C) apoptotic PMN. Human macrophages were seeded in 96-well plates (5×10^4^ cells/well) and incubated with vehicle (PBS containing 0.1% ethanol), MaR1 or 13R,14S-diHDHA (PBS^+/+^, pH 7.45, 37°C, 15 min). (B) FITC-labeled zymosan (5×10^5^ particles/well) or (C) fluorescently labeled apoptotic PMN (1.5×10^5^ cells/well) were added and cells incubated for an additional 60 min (pH 7.45, 37°C). Non-phagocytosed zymosan or apoptotic PMN were washed, extracellular florescence quenched and phagocytosis quantified. Results are mean ± SEM. n = 3 separate human macrophage preparations (*P<0.05, **P<0.01, ****P<0.0001 *vs*. vehicle; #*P*<0.05, ###*P*<0.001 *vs*. MaR1).

We next assessed the potential anti-inflammatory actions of the novel macrophage product isolated by investigating its ability to regulate leukocyte response *in vivo*. Mouse peritonitis was initiated by *i.p*. injection of zymosan (0.1 mg/mouse) [11]. Peritoneal lavages were collected at 4 h and PMN were enumerated by light microscopy and flow cytometry. Systematic administration of 1 ng/mouse of the 13R,14S-diHDHA prior to initiating peritonitis reduced PMN infiltration into the peritoneum by ∼40%; similar values were obtained for MaR1 (**Fig. 6a**). In addition, 13R,14S-diHDHA also enhanced human macrophage phagocytosis of zymosan by 90% at a concentration as low as 10 pM, compared to 60% maximal enhancement by MaR1 at 10 nM (**Fig. 6b**). However, with human apoptotic PMN and macrophages, the MaR1 proved more potent than 13R,14S-diHDHA at enhancing efferocytosis.

## Discussion

**Figure 7:**
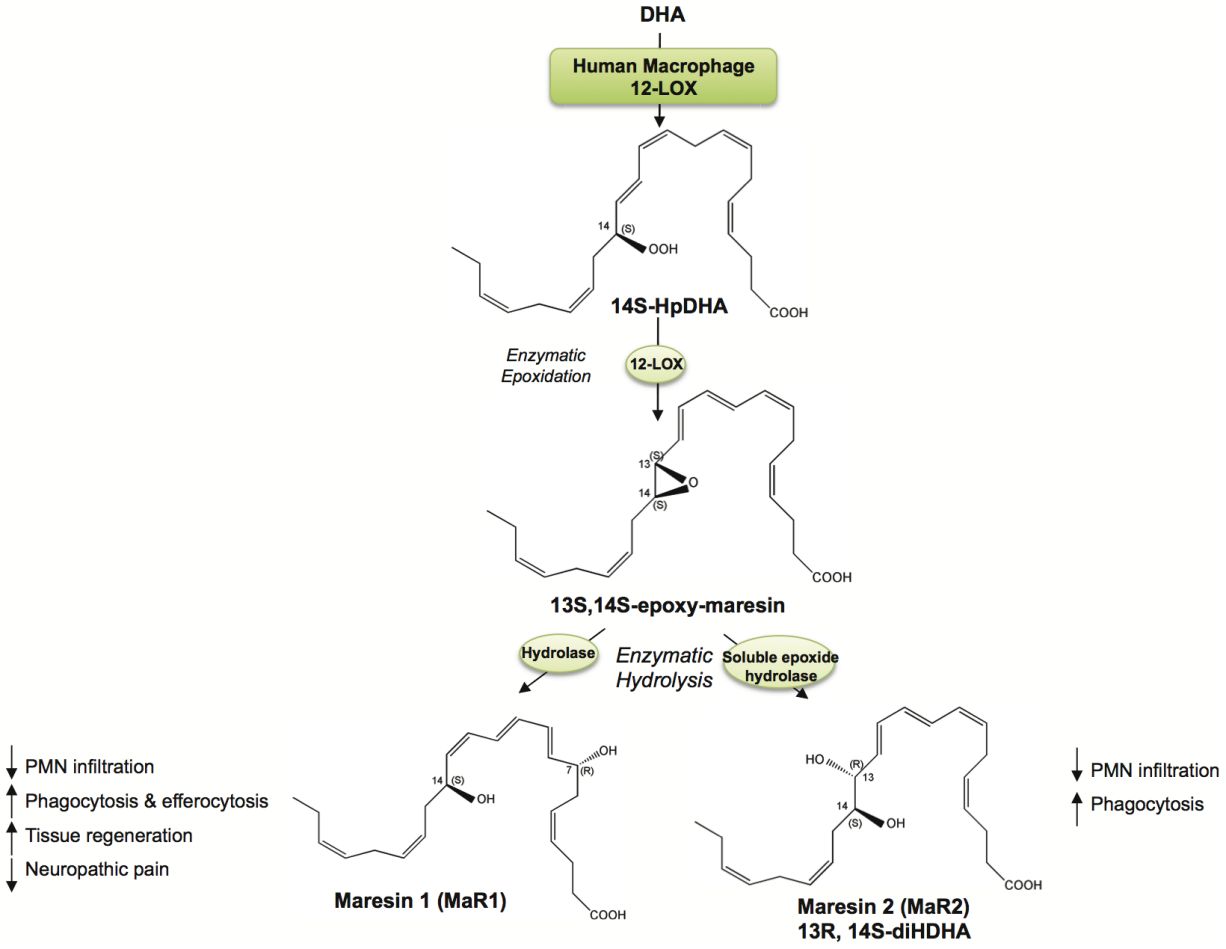
Proposed biosynthetic scheme for MaR1 and MaR2. Human macrophage 12-LOX converts DHA to the 13S,14S-epoxy-maresin intermediate, and via soluble epoxide hydrolase this intermediate is converted to MaR2. See text for details and stereochemical assignments.

In the present manuscript, we identified and cloned the human macrophage 12-LOX involved in the biosynthesis and bioactive maresin metabolome, and found a new member of the maresin family produced from DHA. The human macrophage 12-LOX converted both AA and DHA with essentially equivalent efficiency to produce the hydroperoxy products, respectively, that were predominantly in the carbon 14 with S configuration (98% S). A new 13R,14S-diHDHA was identified from human macrophages that displayed potent anti-inflammatory and pro-resolving actions. Production of 13R,14S-diHDHA involved the initial oxygenation at C-14 followed by 12-LOX-catalyzed epoxidation and subsequent hydrolysis via sEH. The proposed biosynthetic schemes of MaR1 and 13R,14S-diHDHA are summarized in **Fig. 7**. Given the potent anti-inflammatory and pro-resolving actions of the new 13R,14S-diHDHA diol and its biosynthesis from the 13S,14S-epoxy-maresin [6], we coined this product as MaR2.

Maresins are biosynthesized by human macrophages 12-LOX from DHA. MaR1 is the first member of this family to be identified [4]. In addition to its anti-inflammatory, pro-resolving [4], tissue regenerative and anti-nociceptive actions [5], [6], MaR1 was recently found to dampen the pro-inflammatory response to organic dust in bronchial epithelial cells [20], and attenuates mouse colitis [21]. MaR1 was also identified in human synovial fluid from rheumatoid arthritis patients [22]. Another related 12-LOX-derived product, 14S,21R-diHDHA, was shown to enhance wound healing and rescues mesenchymal stem cell function in diabetes and renal ischemia/reperfusion injury [23]–[25]. In the present manuscript, we identified a novel member of the maresin family, namely 13R,14S-diHDHA, coined MaR2, that is produced by the human 12-LOX and sEH in human macrophages. MaR2 exhibited similar potency to MaR1 in limiting PMN infiltration, but had an apparent optimal concentration 2–3 log orders lower than MaR1 in enhancing human macrophage phagocytosis of zymosan (**Fig. 6**). MaR2 also enhanced human macrophage uptake of apoptotic PMN, but was less potent than MaR1. These are the key defining bioactions of a pro-resolving mediator [1], [2].

Also, we confirmed that the activities of DHA oxygenation and epoxidation recently identified [4], [6] in human macrophages originated from 12-LOX. The nucleotide sequence of the human macrophage 12-LOX was in accordance [cf. ref. 15] with human platelet type; see **Figure S2**. Human epidermis also has been shown to express the same platelet type of 12-LOX [26]. Human 12-LOX oxygenates AA at C-12 with S chirality [27]. In the present report, we found that human macrophage 12-LOX oxygenated DHA at the C-14 with a predominant S chirality (**Fig. 3**). The 14S-HDHA profiling obtained here was consistent with that from primary macrophages and DHA [4]. The two-carbon position shift in DHA compared to AA is consistent with the tail-first substrate access [28]. In addition, human macrophage 12-LOX displayed an epoxide synthase activity converting 14-hydroperoxy-4Z,7Z,10Z,12E,16Z,19Z-docosahexaenoic acid (14-HpDHA) to 13S,14S-epoxy-maresin, as demonstrated by methanol trapping [6]. This product is a central intermediate in maresin biosynthesis and production of both MaR1 and MaR2 (**Fig. 7**). 12-LOX also biosynthesizes lipoxins from leukotriene A_4_ (LTA_4_) [29], [30] and this enzyme is susceptible to suicide inhibition by epoxides, such as with LTA_4_ [30] or 13S,14S-epoxy-maresin [6]. Interestingly 13S,14S-epoxy-maresin only inhibits conversion of AA by 12-LOX, while conversion of DHA appeared unaltered [6], thereby suggesting that 13S,14S-epoxy-maresin exerts a feed-forward action on the maresin pathway and hence in resolution of inflammation.

Macrophages play an indispensable role in resolution of inflammation and re-establishment of homeostasis. M1 macrophages, stimulated by IFN-γ LPS and GM-CSF, resist to microbial invasion and enhance inflammatory response [31]. In contrast, M2 macrophages, differentiated on exposure to IL-4, IL-13 and/or other cytokines as well as hormones, produce decreased level of pro-inflammatory cytokines and promote resolution [31],[32], whereas DCs play a key role in the transition between innate and adaptive immunity [33]. Here, human macrophage 12-LOX initiates biosynthesis of maresins, and more importantly, is responsible for the production of 13S,14S-epoxy-maresin. Of note, 12-LOX mRNA expression levels remain unchanged during differentiation of human monocytes to macrophages, and in macrophages, 12-LOX mRNA is not regulated by overnight stimulations with LPS, multiple cytokines or hypoxia [34]. In agreement with this, in the present investigation, we did not find significant difference of 12-LOX mRNA levels in human monocytes, and M0, M1 and M2 macrophages. However, 12-LOX mRNA levels in mDC were significantly increased after LPS stimulation, when compared with iDC (**Fig.1**). Assessment of 12-LOX protein expression in M0, M1 and M2 macrophages demonstrated significantly increasing 12-LOX protein levels when compared to monocytes (**Fig.1**), suggesting that translational or post-translational regulation mechanism also plays a role in establishing 12-LOX protein level in these cells. Of interest, we also found that mDC possess the highest 12-LOX protein level compared to other monocyte-derived lineages examined herein, suggesting that mDC may be a notable source of maresins that can exert pro-resolving actions during resolution of inflammation.

Given the potent actions of maresins in resolution and the role of human macrophage 12-LOX in maresin biosynthesis, we assessed kinetics of conversion for DHA by 12-LOX, finding that 12-LOX catalyzes AA and DHA with equivalent efficiency (**Table 1**). Enzymatic turnover approaches the maximal rate with incubation of either 50 µM AA or DHA. This is less than the reported critical micelle concentration for either DHA or AA [35]. Hence, increased dietary levels of DHA are likely to switch to maresin production and enhance the resolution phase on the infiltrating macrophages in exudates or by DC at local microenvironments in lymphoid tissues and elsewhere in humans.

## Conclusions

Results obtained in the present studies with recombinant enzymatic co-incubations demonstrated that biosynthesis of 13R,14S-diHDHA involves a sEH subsequent to 12-LOX (**Fig. 5**). Mammalian sEH protein and its activity are present in mononuclear cells and macrophages [16], [17]. Mammalian sEH enzymes catalyze hydrolysis of a broad category of epoxides, including epoxyeicosatrienoic acids [36], LTA_4_ [18] and even hepoxilins [37]. Our finding further demonstrated that 13S,14S- epoxy-maresin is a new substrate for sEH by coincubation of 12-LOX and sEH with DHA, producing a potent new product (**Fig. 5**). Since LTA_4_ is converted by sEH to 5S,6R-diHETE with double bond geometry 7E,9E,11Z [38]. Based on this mechanism, we tentatively assigned the complete stereochemistry of MaR2 as 13R,14S-dihydroxy-4Z,7Z,9E,11E,16Z,19Z-hexaenoic acid (see **Figure 7**). Taken together, we identified and cloned a human macrophage 12-LOX and marked its expression in monocyte lineage. The enzyme matched the human platelet enzyme cloned earlier [15], [39], [40]. We also identified a novel pro-resolving product from MΦ, MaR2, and characterized its biosynthesis involving 12-LOX as well as its bioactions. The present findings provide additional evidence for the macrophage pro-resolving metabolome and their potential in regulating inflammation and stimulating resolution.

## Supporting Information

Figure S1 **Peripheral blood mononuclear cells (PBMC) were isolated from human whole blood by density gradient centrifugation using Histopaque-1077 and Phenotypic differentiations were obtained by culture as described in** **methods** **.** Each lineage was confirmed by flow-cytometry using the surface markers. Results are representative of n = 4.
(PDF)

Figure S2 (A) Two fragments of h12LOX were obtained by using cDNA from M0 as template. Two fragments together cover the entire coding area in h12LOX cDNA with ∼200 bp overlap. (B) Human macrophage 12-LOX cDNA was cloned to pET20b vector and sequenced. The result matches the platelet type of 12-LOX sequence. (C) Expression of 12-LOX protein in macrophages and DCs was detected by western blotting with anti-human platelet 12-LOX antibody. 75 kD bands were present in each cell lineage, and the size is consistent with platelet type.
(PDF)
