# Supplementary figures and images for: Maresin Biosynthesis and Identification of Maresin 2, a New Anti-Inflammatory and Pro-Resolving Mediator from Human Macrophages
Source: PLoS One. 2014 Jul 18;9(7):e102362. doi: 10.1371/journal.pone.0102362 (PMC4103848; doi:10.1371/journal.pone.0102362)

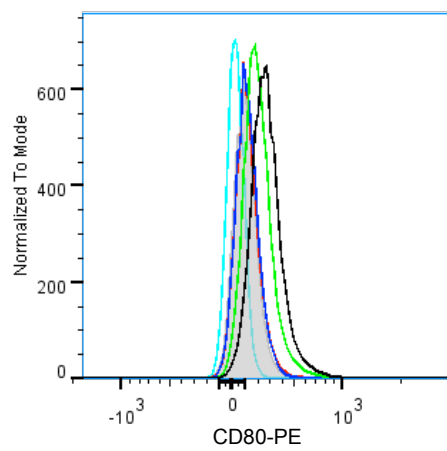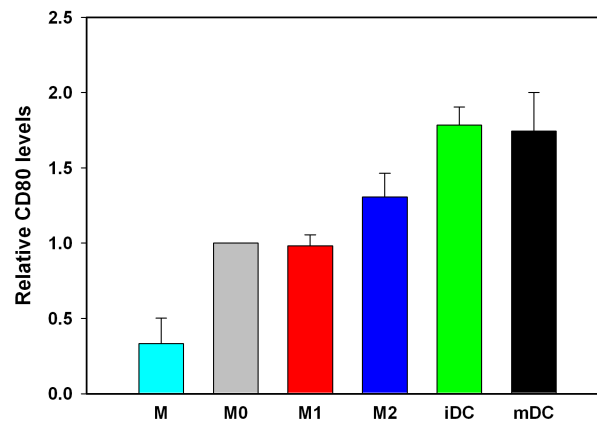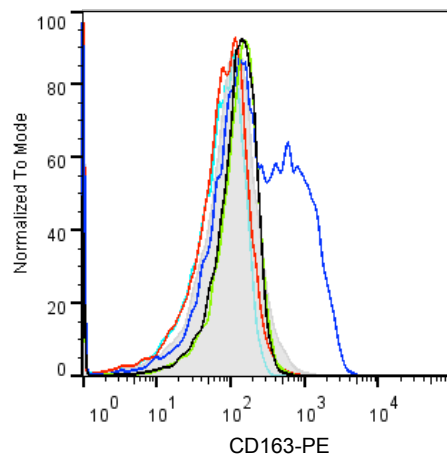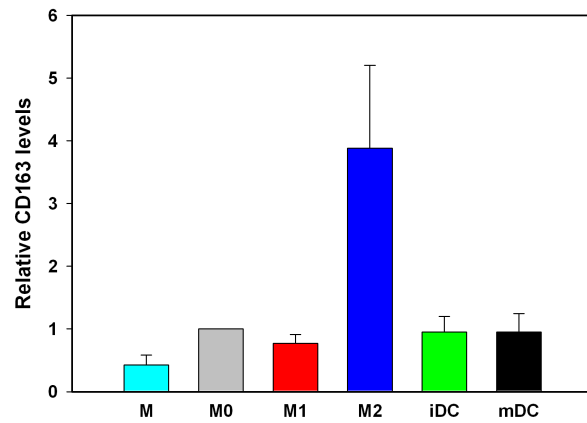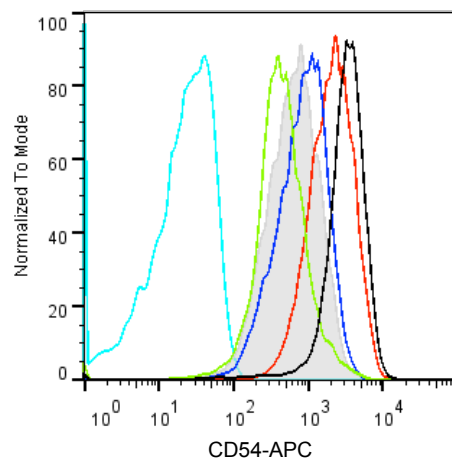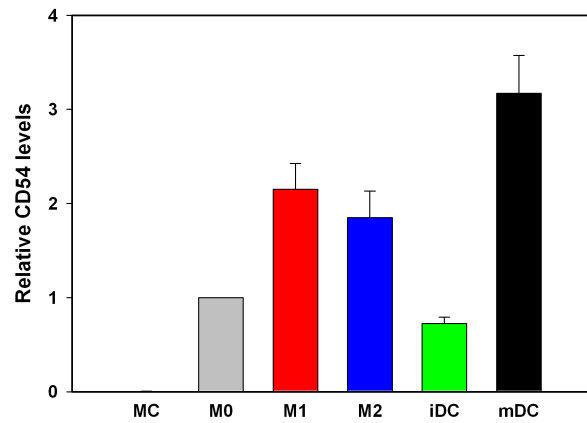

Supplement: Figure S1 — Peripheral blood mononuclear cells (PBMC) were isolated from human whole blood by density gradient centrifugation using Histopaque-1077 and Phenotypic differentiations were obtained by culture as described in methods . Each lineage was confirmed by flow-cytometry using the surface markers. Results are representative of n = 4. (PDF) [file pone.0102362.s001.pdf]
