# Supplementary material for: Maresin Biosynthesis and Identification of Maresin 2, a New Anti-Inflammatory and Pro-Resolving Mediator from Human Macrophages
Source: PLoS One. 2014 Jul 18;9(7):e102362. doi: 10.1371/journal.pone.0102362 (PMC4103848; doi:10.1371/journal.pone.0102362)

A

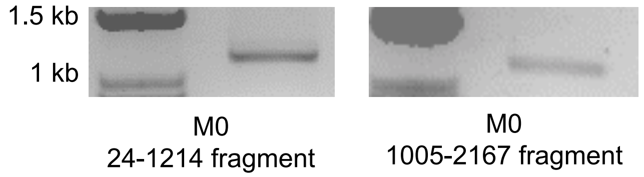

B

ATGGGCCGCTACCGCATCCGCGTGGCCACCGGGGCCTGGCTCTTCTCCGGGT
CGTACAACCGCGTGCAGCTTTGGCTGGTCGGGACGCGCGGGGAGGCGGAGCT
GGAGCTGCAGCTGCGGCCGGCGCGGGGCGAGGAGGAGGAGTTTGATCATGA
CGTTGCAGAGGACTTGGGGCTCCTGCAGTTCGTGAGGCTGCGCAAGCACCAC
TGGCTGGTGGACGACGCGTGGTTCTGCGACCGCATCACGGTGCAGGGCCCTG
GAGCCTGCGCGGAGGTGGCCTTCCCGTGCTACCGCTGGGTGCAGGGCGAGGA
CATCCTGAGCCTGCCCGAGGGCACCGCCCGCCTGCCAGGAGACAATGCTTTG
GACATGTTCCAGAAGCATCGAGAGAAGGAACTGAAAGACAGACAGCAGATC
TACTGCTGGGCCACCTGGAAGGAAGGGTTACCCCTGACCATCGCTGCAGACC
GTAAGGATGATCTACCTCCAAATATGAGATTCCATGAGGAGAAGAGGCTGGA
CTTTGAATGGACACTGAAGGCAGGGGCTCTGGAGATGGCCCTCAAACGTGTT
TACACCCTCCTGAGCTCCTGGAACTGCCTAGAAGACTTTGATCAGATCTTCTG
GGGCCAGAAGAGTGCCCTGGCTGAGAAGGTTCGCCAGTGCTGGCAGGATGAT
GAGTTGTTCAGCTACCAGTTCCTCAATGGTGCCAACCCCATGCTGTTGAGACG
CTCGACCTCTCTGCCCTCCAGGCTAGTGCTGCCCTCAGGGATGGAAGAGCTTC
GGGCTCAACTGGAGAAAGAACTTCAGAATGGTTCCCTGTTTGAAGCTGACTT
CATCCTTCTGGATGGAATTCCAGCCAACGTGATCCGAGGAGAGAAGCAATAC
CTGGCTGCCCCCCTCGTTATGCTGAAGATGGAGCCCAATGGGAAGCTGCAGC
CCATGGTCATCCAGATTCAGCCTCCCAACCCCAGCTCTCCAACCCCAACACTG
TTCCTGCCCTCAGACCCCCCACTTGCCTGGCTCCTGGCAAAGTCCTGGGTCCG
AAATTCAGATTTCCAACTGCACGAGATCCAGTATCACTTGCTGAACACGCAC
CTGGTGGCTGAGGTCATCGCTGTCGCCACCATGCGGTGCCTCCCAGGACTGC
ACCCCATCTTCAAGTTCCTGATCCCCCATATCCGCTACACCATGGAAATCAAC
ACCCGGGCCCGGACCCAACTCATCTCAGATGGAGGAATTTTTGATAAGGCAG
TGAGCACAGGTGGAGGGGGCCATGTACAGTTGCTCCGTCGGGCGGCAGCTCA
GCTGACCTACTGCTCCCTCTGTCCTCCTGACGACCTGGCTGACCGGGGCCTGC
TGGGACTCCCAGGTGCTCTCTATGCCCATGATGCTTTACGGCTCTGGGAGATC
ATTGCCAGGTATGTGGAGGGGATCGTCCACCTCTTCTACCAAAGGGATGACA
TAGTGAAGGGGGACCCTGAGCTGCAGGCCTGGTGTCGGGAGATCACGGAGGT
GGGGCTGTGCCAGGCCCAGGACCGAGGTTTCCCTGTCTCCTTCCAGTCCCAG
AGTCAACTCTGCCATTTCCTCACCATGTGCGTCTTCACGTGCACTGCCCAGCA
TGCCGCCATCAACCAGGGCCAGCTGGACTGGTATGCCTGGGTCCCTAATGCT
CCATGCACAATGCGGATGCCCCCACCCACCACCAAGGAAGATGTGACGATGG
CCACAGTGATGGGGTCACTACCTGATGTCCGGCAGGCCTGTCTTCAAATGGC
CATCTCATGGCATCTGAGTCGCCGCCAGCCAGACATGGTGCCTCTGGGGCAC
CACAAAGAAAAATATTTCTCAGGCCCCAAGCCCAAAGCTGTGCTAAACCAAT
TCCGAACAGATTTGGAAAAGCTGGAAAAGGAGATTACAGCCCGGAATGAGC
AACTTGACTGGCCCTATGAATATCTGAAGCCCAGCTGCATAGAGAACAGTGT
CACCATCTGA

C

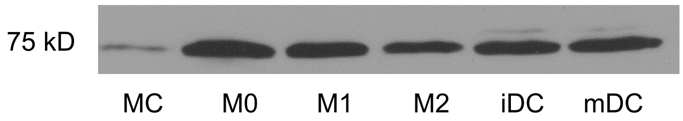

Supplement: Figure S2 — (A) Two fragments of h12LOX were obtained by using cDNA from M0 as template. Two fragments together cover the entire coding area in h12LOX cDNA with ∼200 bp overlap. (B) Human macrophage 12-LOX cDNA was cloned to pET20b vector and sequenced. The result matches the platelet type of 12-LOX sequence. (C) Expression of 12-LOX protein in macrophages and DCs was detected by western blotting with anti-human platelet 12-LOX antibody. 75 kD bands were present in each cell lineage, and the size is consistent with platelet type. (PDF) [file pone.0102362.s002.pdf]
